# Targeted Delivery of Drugs and Genes Using Polymer Nanocarriers for Cancer Therapy

**DOI:** 10.3390/ijms22179118

**Published:** 2021-08-24

**Authors:** Wentao Xia, Zixuan Tao, Bin Zhu, Wenxiang Zhang, Chang Liu, Siyu Chen, Mingming Song

**Affiliations:** School of Life Science and Technology, China Pharmaceutical University, Nanjing 211198, China; 3320031498@stu.cpu.edu.cn (W.X.); 3219030715@stu.cpu.edu.cn (Z.T.); 3220030444@stu.cpu.edu.cn (B.Z.); wenxiangzhang@cpu.edu.cn (W.Z.); changliu@cpu.edu.cn (C.L.)

**Keywords:** polymer nanocarriers, cancer therapy, drug delivery

## Abstract

Cancer is one of the primary causes of worldwide human deaths. Most cancer patients receive chemotherapy and radiotherapy, but these treatments are usually only partially efficacious and lead to a variety of serious side effects. Therefore, it is necessary to develop new therapeutic strategies. The emergence of nanotechnology has had a profound impact on general clinical treatment. The application of nanotechnology has facilitated the development of nano-drug delivery systems (NDDSs) that are highly tumor selective and allow for the slow release of active anticancer drugs. In recent years, vehicles such as liposomes, dendrimers and polymer nanomaterials have been considered promising carriers for tumor-specific drug delivery, reducing toxicity and improving biocompatibility. Among them, polymer nanoparticles (NPs) are one of the most innovative methods of non-invasive drug delivery. Here, we review the application of polymer NPs in drug delivery, gene therapy, and early diagnostics for cancer therapy.

## 1. Introduction

Cancer is still the second leading cause of death globally, and its death toll exceeds the combined deaths from human immunodeficiency virus/acquired immunodeficiency syndrome, tuberculosis, and malaria [1,2,3,4]. In 2020, GLOBOCAN estimated that 19.3 million new cancer cases and almost 10.0 million cancer deaths occurred worldwide [5,6]. Cancer includes a series of diseases caused by the uncontrolled growth of malignant cells, which may invade or spread to other parts of the body [7,8]. Thus far, many strategies have been developed for cancer treatment, including surgery [9,10], radiation therapy [11,12,13], chemotherapy [14,15,16,17], targeted therapy [18,19,20], hormonal therapy [21,22], and immunotherapy [23,24], or a combination of these options [25,26]. As a result of all these treatments, the incidence of cancer has declined slightly over the past decade.

However, traditional therapies are only effective for some early malignant tumors [27,28,29], and the main reasons for eventual failure of tumor treatment are metastasis [30], recurrence [31], heterogeneity [32], chemotherapy resistance [33], and avoidance of immune surveillance [34]. Chemotherapy is mainly achieved by using chemotherapeutic drugs to kill cancer cells [35,36]. As a means of systemic treatment, the drug often circulates throughout most of the organs and tissues of the body with the blood, which can cause damage to other healthy tissues and organs [37,38]. However, chemotherapy has a reliable effect on some tumors that tend to spread throughout the body and on metastatic tumors [39,40,41]. The classic chemotherapeutic drugs (doxorubicin (Dox) [42], vemurafenib [43], and paclitaxel (PTX) [44]) are still the mainstay of current treatment, but they are limited by narrow treatment indicators, significant toxicity, and frequent acquired drug resistance. Traditional chemotherapy interferes with DNA synthesis and mitosis, leading to the death of fast-growing and dividing cancer cells [16,45,46]; however, these drugs are non-selective and can damage healthy normal tissues, causing serious complications and undesirable side effects, such as loss of appetite and nausea. In fact, the severe adverse effects of chemotherapy drugs on healthy tissues and organs are the main reason for the high mortality of cancer patients.

Further research into the pathogenesis of cancer has led to new treatment options, including targeted therapy [47,48] and immunotherapy [49,50]. Targeted chemotherapy mainly uses molecular targeted drugs to block specific molecules and metabolic pathways in tumor cell growth and proliferation. Compared to other types of cancer treatments, targeted therapy can achieve the greatest therapeutic effect and lowest toxicity. In particular, the more targeted the drug, the lower the possibility of drug resistance. The clinical success of immunotherapy has revolutionized the treatment of a variety of advanced malignant tumors [51,52]. However, most patients do not benefit from existing immunotherapies, and many patients experience immune-related adverse events [53]. It is generally believed that the development of new anticancer drugs has greatly improved the survival and quality of life of cancer patients. However, in many cases, these drugs show a good response during initial treatment, but later on in the treatment, the efficacy of the drugs decreases and can lead to cancer recurrence. This phenomenon is called acquired drug resistance and is a major problem in the treatment of cancer [54,55,56]. The formation of drug resistance is mainly due to a kind of internal resistance that develops within tumor cells [57,58,59]. A specific cell membrane transporter changes the drug transport and pumps the drug out of tumor cells [60]. In addition, the gradual acquisition of specific heredity and epigenetics in cancer cells during the treatment process greatly contributes to acquired drug resistance [54]. Drug resistance is defined as a decline in the efficacy and potency of a drug in order to limit treatment, ultimately leading to failure in the treatment of the disease [61]. Tumors such as kidney cancer [62], hepatocellular carcinoma [63], and malignant melanoma [64] often respond well to chemotherapy in the early stage, but become unresponsive in later stages of treatment due to the development of acquired drug resistance.

In order to solve these problems, we urgently need to develop new treatment methods to help improve clinical efficacy. The emergence of nanotechnology has had a profound impact on the clinical treatment of tumors, which has promoted the rapid development of targeted therapy [64,65,66], combined drug therapy [67,68], and early tumor diagnosis [69]. Among them, NDDSs have become a research hotspot at the interface between nanotechnology and biomedicine, because of their efficient loading, targeted delivery, controlled release, and other functions for drugs, and show promise in biomedical applications [70,71]. For example, silicon-based nanomaterials [72], polymers [73], liposomes, and metal NPs [74] are designed to deliver anticancer drugs to tumor tissues. In various NDDSs, polymer-functionalized nanomaterials have attracted widespread attention as excellent candidate materials for therapeutic drug delivery, especially based on their multivalent binding ability and ideal biocompatibility [75,76]. In this paper, we discuss various types of polymer NPs and focus on their applications in traditional chemotherapy, immunotherapy, gene therapy, and combination therapy.

## 2. Nanocarriers

### 2.1. Physical and Chemical Properties of Nanocarriers

In recent years, NP delivery systems as drug delivery carriers have aroused extensive research interest in the field of cancer precision medicine. NPs are particle dispersions or solid particles with a particle size in the range of 10–1000 nm [77]. Depending on the material, the NPs include lipids (liposomes) [78], polymers (artificial synthetic polymer NPs and natural polymer NPs) [79], inorganic NPs (silicon NPs) [80], organic compounds (carbon nanotubes) [81], and metal NPs (gold NPs, silver NPs, magnetic NPs, etc.) [82]. Most studies have suggested that the NP delivery systems described above can be used to alter and improve the pharmacokinetic and pharmacodynamic properties of various types of drug molecules [83,84,85]. The main reason is that NP delivery systems prolong the half-life of the drug in vivo, limit the entry of the drug into normal histiocytes, and regulate the release of the drug in the target organ tissue at a controllable and continuous rate. As ideal nanodrug delivery systems, in addition to being able to specifically target drug delivery to tumor tissue, NPs must also have a long circulatory function that is not easily recognized by the phagocytic cells of the reticuloendothelial system. The physical and chemical properties of NPs consist primarily of the following: Size, surface charge, shape, composition, and modification of surface groups [86,87].

The particle size and particle size distribution of NPs are two of the most important characteristics of drug delivery systems [88]. They determine the distribution, half-life, toxicity, and targeting ability of NP systems in vivo [89]. In addition, they affect the drug loading, release, and stability of NPs. The release of a drug is mainly affected by the size of the particle. Smaller particles have a larger surface area, so most of the drug contained in the carrier attaches to or near the surface of the particle, resulting in rapid drug release [90]. It is known that the clearance rate of very small NPs can be faster, and most of these NPs end up in the liver and spleen; hence, the usage of these targeted NPs is impractical and ineffective. On the other hand, micro-carriers are too large to be administered through small capillaries. Therefore, choosing the right material and particle size is another important aspect in the selection of proper targeting NPs to treat cancer. Nowadays, researchers can rely on the preparation process to adjust the size of NPs according to actual needs [91].

### 2.2. Challenges and Strategies of NPs as Drug Delivery Carriers in Cancer Therapy

Nanocarriers have important advantages, including adjustable physical and chemical properties, ease of production, scalability, and stability during storage [92]. Compared with other delivery vehicles, these are the basic factors for expanding clinical applicability. The fundamental challenge of using NPs for successful gene therapy lies in biological barriers, targeted therapy, and safety [93]. Understanding these obstacles in cancer gene therapy in detail and developing methods to bypass them are critical to realizing NPs’ ultimate potential.

#### 2.2.1. Biodistribution and Barrier Properties

The biodistribution of NPs includes the two interrelated challenges of nano-drug particles gathering in unwanted locations as well as targeting target locations, and their guidance or misdirection by barrier properties [94]. The biodistribution and final particle activity largely depend on the protein corona that forms initially upon contact of the nanomaterial with the body’s various biological components [95]. Change in the protein corona relies heavily on the physicochemical properties of the polymer NPs and circulation time. In addition, barrier properties to drug delivery can prevent the successful accumulation of nano-therapeutic drugs at the disease site, limiting effective responses to disease processes from cancer to inflammation. These obstacles include arrangement and subsequent isolation of the mononuclear phagocytic system (MPS), nonspecific distribution, blood/vascular flow restriction, pressure gradients, cellular internalization, escape from endocrine and lysozyme chambers, and drug excretion pumps [96]. Although a large number of research efforts are aimed at incorporating multiple functions and functions into the overall nanoparticle design, many of these strategies have failed to adequately address these obstacles [97]. Traditional NPs need to be reimagined to successfully resolve these obstacles that hinder drug delivery. Unless nanodrugs are designed with consideration of most (if not all) biological barriers encountered where NPs can escape after entering the body, these obstacles will continue to limit their clinical application in tumor treatment.

In order to overcome the challenges of mass transport across barrier properties and biodistribution, receptor targeting ligands and peptides have been used as mechanisms for the direct transport of therapeutic NPs, as well as cell-mediated drug transport. In recent years, the surface characteristics of NPs have been identified as important factors in determining their lifetime and fate, and are related to their capture by macrophages during the cycling process [98,99]. Ideally, NPs with a hydrophilic surface can more easily escape capture by macrophages. At present, the surface of NPs is generally modified, for example by coating the surface of NPs with hydrophilic polymers (such as poly(ethylene glycol) (PEG)) to prevent the formation of a proteins corona in order to extend the circulation period of NPs in vivo [100,101,102]. Gao et al. found that the larger the PEG modification on the surface of gold NPs, the smaller the particle size and the lower the plasma protein adsorption capacity, which inhibited the formation of a “proteins corona” and enhanced targeting ability mediated by the arginine–glycine–aspartate (RGD) peptide [103]. The strategy of functionalizing NPs with PEG or PEGylation is mainly derived from the observation that the circulatory life of NPs is low after intravascular administration [104]. PEGylation involves grafting PEG onto the surface of NPs, where ethylene glycol units are closely associated with water molecules to form a moisturizing layer [105]. This moisturizing layer in turn hinders protein adsorption and subsequent removal of MPS. In addition, researchers have recently developed a biomimetic particle coating composed of cell membranes separated from corresponding cells (red blood cells, white blood cells and tumor cells, etc.) [65,106,107], which enhanced the immune escape of NPs and extended the lifetime of drugs in the body. When the cell membrane surface was functionalized, amount of protein (IgG and albumin) adsorbed on the particle surface was reduced by over 10-fold [108]. Therefore, this biomimetic coating strategy significantly reduced the uptake of particles by macrophages, especially when the coating originated from the same donor species. Consistently, a low accumulated degree of functionalized particles in the liver has been observed when testing such a platform in a mouse model by systemic administration [109].

#### 2.2.2. Tumor Targeting

Heterogeneity, high metastasis, and invasiveness within the tumor, as well as the lack of clear tumor surface markers, further hinder the development of efficient targeted drug/gene delivery [110]. At present, there are many options for achieving tumor-targeted gene delivery. For example, our previous work mainly focused on passive targeting through the enhanced permeability and retention (EPR) effect or through cancer cell-specific ligands (such as antibodies, peptides, and surface mountants) [111,112]. Active targeting enhances the targeting ability of NPs to maximize tumor distribution and deep tissue penetration. In recent years, targeting the tumor microenvironment has become a promising strategy to overcome tumor resistance, prevent metastasis, and improve the efficacy of gene therapy [113]. NPs have been designed to be sensitive to low pH and high metalloproteinase-2 levels in the tumor microenvironment and locally regulate angiogenesis and hypoxia. Recently, Wang et al. have shown that pH-sensitive drug delivery systems can deliver and release drugs within cancer cells and/or in a more acidic microenvironment inside cancer cells [114]. Collectively, given the heterogeneity of tumors, rational design and evaluation of NPs are necessary.

#### 2.2.3. Safety of Nanocarriers

Safety is another important concern for translational medicine. The main goal of designing NPs as drug delivery systems is to control the particle size, surface properties, and release of pharmaceutically active substances, so that the drug can be accurately delivered to the pathological site under the best time and dosage regimen for treatment. Liposomes have become one of the first nanocarriers used in clinical treatments due to their unique advantages [115]. Liposomes can effectively protect the drug from degradation, target the site of action, and reduce the toxicity or side effects, but their application is limited by inherent problems such as low encapsulation efficiency, rapid seepage of water-soluble drug during in vivo circulation, and poor stability [116]. Researchers have found that polymer NPs are more likely than liposomes to help stabilize drugs (proteins and genes) and have useful controlled release properties [66,117,118]. In addition, the potential toxicity and safety issues of most nanomaterials are the main factors limiting their clinical applications [119]. In vivo, these materials may cause immune responses and cytotoxicity, and may have the ability to clear in the internal organs [120]. At the same time, nanomaterials are often endowed with the ability to cross various barriers and interact with different cellular components such as proteins, lipids, and genetic materials. Therefore, a comprehensive assessment of the safety of nanomaterials will help their application in the clinical treatment of tumors [121]. Polymer NPs exhibit properties such as easy degradation, low immunogenicity, and non-toxicity, which have attracted much attention from researchers [122]. For example, the biodegradable synthetic polymers commonly used in drug delivery applications, namely PLA and PLGA, have been approved by the US Food and Drug Administration (FDA) [123,124] because of their confirmed safety and biocompatibility and low levels of immunogenicity and toxicity, and their degraded oligomers in the body are easily excreted through a common metabolic pathway [125].

### 2.3. Polymer NPs

**Figure 1:**
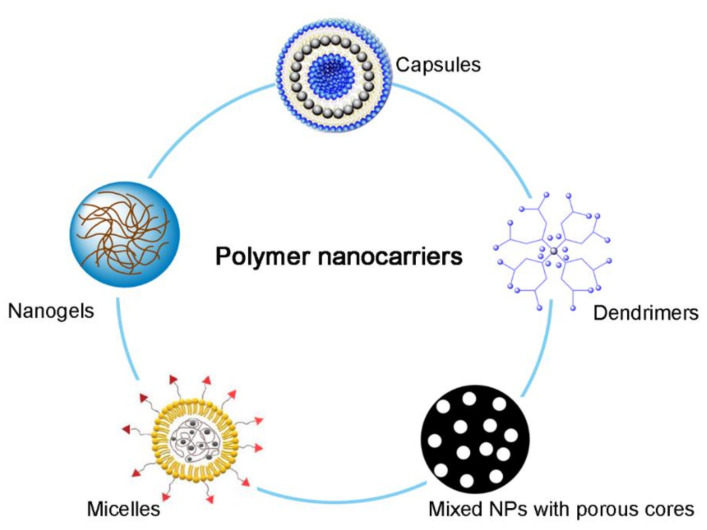
The different types of polymer nanocarriers-based drug delivery for cancer therapeutics [136,137].

Further research on polymer NPs is of great significance to researchers in the fields of science and medicine. Polymer NPs play a central role in a variety of applications, such as drug delivery, medical imaging, and the early detection of disease [126,127,128]. Polymer NPs are particles obtained from natural (chitosan [129], sodium alginate [111] and cyclodextrin [130], etc.), semi-synthetic or synthetic polymers (poly(lactic-co-glycolic acid)-poly(ethylene glycol) (PLGA-PEG) [131], *N*-(2-hydroxypropyl)methacrylamide (HPMA) [132] and poly(acrylamide) (PAM) [133]). Polymer nanosystems are produced by the polymerization of many monomer units. Under certain conditions, they can be organized and self-assembled to the size of 10–200 nm. Polymer nanocarriers are generally divided into five types, namely micelles, nanogels, capsules, dendrimers, and mixed NPs with porous cores [134]. Polymer NPs have a relatively large surface area, which facilitates the surface modification of functional groups and increases the specific distribution of drugs in the body [135]. Moreover, the controlled release properties of polymer NPs and their protective effects on compounds make these NDDSs very advantageous, especially in the field of drug delivery applications. Currently, various polymers have been used in NP drug delivery research to increase the therapeutic benefits while minimizing side effects (Figure 1). Overall, the advantages of polymer NPs as carriers include controlled release, protection, and specific targeting ability of drug molecules.

## 3. Application of Polymer Nanocarriers in Tumor Targeted Therapy

### 3.1. Chemotherapy Based on Polymer Nanocarriers

Chemotherapy is the most common treatment for cancer [14]. As is well-established, chemotherapeutic drugs can be divided into alkylating agents (nimustine, cyclophosphamide, glycosyl mustard, etc.) [138], antimetabolites (deoxyfluoroguanosine, amcitabine, 5-fluorouracil, etc.) [139], antitumor antibiotics (actinomycin D, Dox, and pelomycin) [140], antitumor plant and animal ingredients (hydroxycamptothecin, PTX, etc.) [141], antitumor hormones (atamitan, anastrozole, and nolvadex, etc.) [142], etc. Unfortunately, the current clinical application of antitumor chemotherapeutic drugs has led to unforeseen toxicity and side effects, thus limiting the drug dosage and use. When they kill tumor cells, they also damage normal tissue cells [143]. Among them, the destruction of the body’s immune system caused by the killing of lymphoid tissue cells further aggravates the development of cancer. In addition, due to its toxic and side effects, chemotherapy sometimes has complications such as infection and bleeding. To solve these problems, polymer NPs, as chemotherapeutic drug delivery systems, not only have high drug loading capacities but also can target the delivery of drugs to cancer tissues and control the release of drugs [144]. Polymer NDDSs can improve drug hydrophilicity and encapsulation efficiency, thereby protecting fragile molecules from early degradation/metabolism and prolonging the half-life of the drug during the metabolic cycle. As shown in Table 1, these drug delivery systems can specifically deliver chemotherapeutic drugs to tumor sites and reduce toxicity and side effects.

Fang et al. found that Dox-loaded dextran-based nanocarriers are an effective drug delivery system for the treatment of malignant lymphoma with reduced cardiotoxicity [158]. First, dextran reacts with the monomer methyl-acrylate under the catalysis of cerium ammonium nitrate, and then the crosslinking agent diallyl-disulfide is added to form a new type of nanocarrier [159]. Finally, Dox is covalently bonded to the nanocarrier through a hydrazone bond. This novel drug delivery system not only has a high drug loading capacity and pH sensitivity, but can also reduce cell resistance. The emergence of multidrug resistance in cancer treatment is a huge challenge that limits drug efficacy, thus leading to the failure of many chemotherapy drugs in clinical treatment. As far as polymer NPs are concerned, Cuvier et al. [160], Nemati et al. [161], and Verdière et al. [162]. showed that Dox-loaded polyalkylcyanoacrylate NPs (PACA NPs) can overcome multidrug resistance. In 1997, Verdière et al. used Dox-loaded PACA NPs to overcome multidrug resistance in vitro. They found that the main reason for PACA NPs’ ability to reverse multidrug resistance was that the NPs adsorbed onto the surface of tumor cells and then released their coated drugs, thereby forming a local highly concentrated gradient drug concentration around the tumor cells. At the same time, the degradation and release of polycyanoacrylic acid and other compounds by NPs may have interacted with Dox, which may have promoted the accumulation of Dox in tumor cells by overcoming the transmembrane potential. Moreover, Vlerken et al. found that polymer NPs can be used to modulate intracellular ceramide to overcome multidrug resistance in cancer [163]. In 2015, Yuan et al. proposed self-assembled nanodrugs in cells as a new strategy for overcoming multidrug resistance [164]. They designed a taxol derivative (Ac-Arg-Val-Arg-Arg-Cys(StBu)-Lys(taxol)-2-cyanobenzothiazole (CBT-Taxol)) through a biocompatible condensation reaction. The results showed that the lethality of CBT-Taxol to taxol-resistant HCT 116 cancer cells was significantly higher than that of free taxol. Furthermore, CBT-Taxol had a more lasting effect on tubulin condensation, possibly due to its ability to slowly release taxol in tumor cells. Therefore, the construction of intracellular self-assembled nanodrugs may be a new and optimal strategy to overcome drug resistance. At present, many NP-based drug delivery systems have been developed to overcome drug resistance by increasing cell uptake and rapid drug release to increase the intracellular drug concentration. Wang et al. designed a polypeptide dendritic copolymer NP to encapsulate and target Dox and to overcome multidrug resistance by regulating the lysosomal pathway of breast cancer cell apoptosis [165]. Mu et al. developed self-assembled NPs based on chitosan grafted with cholesterol hemisuccinate to enhance the absorption of docetaxel by multidrug-resistant cancer cells [166]. In 2021, a novel type of folic acid-modified chitosan-silica NPs were used to co-deliver PTX and P-shRNA [167]. These NPs could effectively protect P-shRNA from degradation and exhibited pH-responsive drug release behavior. As a targeting ligand, folic acid could improve the uptake efficiency of NPs by multidrug-resistant breast cancer cells. In addition, these NPs showed excellent P-shRNA release ability in cells, and effectively silenced the expression of the target gene P-gp, thereby reducing the multidrug resistance to the PTX. Therefore, polymer nanocarriers can help to reduce the emergence of multidrug resistance for chemotherapeutic drugs in clinical treatment, can greatly improve the clinical efficacy of chemotherapeutics, and can bring about a new dawn for patients.

### 3.2. Gene Therapy Based on Polymer Nanocarriers

Gene therapy is a technology that treats or cures diseases by modifying a person’s genes. Gene therapy can work through the following mechanisms: (i) replacing disease-causing genes with healthy genes; (ii) inactivating disease-causing genes; (iii) introducing new or modified genes into the body to help treat diseases [168,169]. There are many types of gene therapy products, including plasmid DNA, viral vectors, bacterial vectors, gene editing technology, and patient-derived cell gene therapy products [170]. Among them, gene editing refers to the process of making tiny, controllable changes to the DNA of organisms, mainly using the CRISPR/Cas9 method [171]. Although advances have been made in various nucleotide-based therapies, the low efficiency of targeted tissue or cell delivery has limited the clinical application of gene therapy [172]. In the blood circulation, small nucleic acid molecules are easily degraded by enzymes in plasma. In vivo fluids, the phosphate bonds of nucleic acids, are gradually broken by exonuclease and then cleared by glomerular filtration, and are excreted from the body with urine. Gao et al. have also found that, in the extracellular environment, the half-life of naked small nucleic acid molecules ranges from a few minutes to a few hours, so they cannot be enriched in large amounts to target cells, thereby reducing the bioavailability of the drug and leading to poor efficacy [173]. Based on these problems, we urgently need a new delivery vehicle to enhance the therapeutic response of gene agents (siRNA [174], miRNA [175], mRNA [176], and CRISPR/Cas9 [177]) in the system environment. Given the high instability of naked nucleic acid in systemic circulation, the poor permeability of biofilm, and the ease of missing the target, along with other shortcomings, the design of an appropriate carrier must have the following characteristics: (i) a high encapsulation rate for nucleic acid drugs; (ii) the ability to protect the stable structure of nucleic acid molecules; (iii) accurate delivery of nucleic acid drugs to target tissues and cells.

In recent decades, nanotechnology has made major breakthroughs in the development of safe and efficient gene vectors (Table 2). Compared to viral vectors, nanocarriers not only have good biosafety but can also deliver gene agents to target cells with high efficiency [178]. In addition, NDDSs can maintain their functions while improving the bioavailability of drugs and reducing off-target effects. Among existing nanocarriers, polymer nanocarriers are attracting a great deal of attention due to their non-toxicity, low immunogenicity, and high biocompatibility. RNA interference (RNAi) is a promising technique for regulating tumor genes in cancer therapy [179]. Effective cancer treatment using RNAi requires efficient delivery of siRNA and silencing of target genes in cancer cells. NDDSs are a relatively convenient way to deliver therapeutic siRNA to solid tumors, including tumor metastasis. However, there are multiple obstacles to delivering therapeutic siRNA to the cytoplasm of cancer cells. To overcome the challenges of current siRNA delivery vehicles, researchers have designed many interesting and increasingly complete drug delivery systems. Johan et al. prepared poly(β-amino ester)s (PBAE) through a two-step method and designed a bioreducible PBAE–siRNA NP for the systematic delivery of siRNA in vivo to brain tumors [180]. NPs based on PBAE have high in vitro permeability, and the positively charged surface improves siRNA loading efficiency and can remain stable in the presence of serum proteins. In addition, the diameter of PBAE NPs is approximately 57 nm, small enough to cross the blood–brain barrier (BBB) to reach the glioma site and specifically inhibit the expression of target genes. Li et al. reported a dual supramolecular nanocomposite composed of α-cyclodextrin-modified hyaluronic acid and an azobenzene-modified diphenylalanine derivative with a positively charged imidazole group [181]. Such nanocarriers can bind to siRNA through electrostatic interactions, and effectively deliver them to cancer cells in order to inhibit their growth. In addition, the azobenzene double bonds of NDDs are isomerized under ultraviolet radiation (365 nm), resulting in the disintegration of the NPs to release siRNA, while showing good cytotoxicity toward cancer cells. Li et al. believed that a new drug delivery system was expected to overcome the shortcomings of the high-density positive charges of gene transfection reagents, damaging the membranes and cells of normal cells, thereby providing a promising method for gene delivery. Ashley et al. have shown that most NPs are recognized and eliminated by the immune system, limiting the bioavailability of gene agents [182]. Biomimetic NPs modified with active cell membranes are now one of the most attractive nanostructures. The invisibility of cell membranes allows biomimetic NPs to be altered and functionalized with self-awareness and targeting capabilities to dilate blood circulation and avoid immune capture. Chen et al. designed a cancer cell membrane cloaking NP for the targeted co-delivery of Dox and programmed death-ligand 1 (PD-L1) siRNA [183]. Cancer cell membrane-covered polymer NPs show good internalization of self-recognition, which can effectively camouflage the nanocarrier while also having multiple membrane antigens and surface functionalization.

Therefore, the combination of gene therapy and a nanocarrier-mediated drug delivery system holds important prospects for future cancer treatment. Furthermore, gene therapy offers a promising strategy for cancer treatment by specifically targeting oncogenes. More interestingly, the above strategies can also be combined with NP-mediated imaging methods to safely track and identify the biodistribution of nanomedicines. At the same time, the emergence of polymer nanocarriers has improved the serious challenges of toxicity and immunogenicity posed by other types of nanocarriers, and has broadened the clinical application of nanocarriers in cancer therapy.

### 3.3. Immunotherapy Based on Polymer Nanocarriers

In the past decade, cancer immunotherapy has received great attention. Cancer immunotherapy mainly uses immune checkpoint inhibitors, agonists, antigens, and chimeric antigen receptor T cells to activate the patient’s innate and adaptive immune system to combat tumor cells [189,190]. Unlike other oncologic therapies (such as chemotherapy, radiotherapy, and surgery), immunotherapy aims to restore the antitumor activity of the immune system and use the patient’s own immune system to attack abnormal cells, thereby improving efficacy and reducing missed targets in the treatment of advanced malignant tumors [191]. In recent years, cancer immunotherapy has achieved some significant clinical successes, including cancer vaccines obtaining FDA approval, and immune checkpoint blockade (ICB) [192], adoptive cell transfer (ACT) [193], monoclonal antibody (mAbs) therapy [194], and chimeric antigen receptor (CAR) T cell therapy with programmed cell death 1 (PD-1) [195] or its ligand (PD-L1) [196] as immune checkpoint inhibitors have shown promise. However, the inherent limitations of conventional immunotherapy are the difficulty of precise dose control, insufficient tumor tissue enrichment, and partial immune response silence. This has resulted in the overall response rate of patients still being less than 30%, accompanied by immune-related adverse events (enteritis, pneumonia, hepatitis, myocarditis, and neurotoxic effects) [197].

Therefore, there is an urgent need to improve current cancer immunotherapies, and Ahmed et al. found that NP-based methods can improve their ability to enhance T cell activation on tumor cells and improve their antitumor efficacy with minimal toxicity [198]. Effective control of the release of immune agonists or adjuvants is an important way to avoid the attacks on normal tissues and organs caused by excessive immune activation. Interestingly, studies have found that immunomodulators are encapsulated in biodegradable polymers, such as PLGA, where they are slowly released as the polymer is degraded [199]. Moreover, polymer nanomaterials have been confirmed in numerous studies to have good biocompatibility, easy degradation, and non-toxicity (Table 3).

Nahal et al. developed an approach to protein NP (pNP) engineering based on reactive electrospraying and controlled the particle size, elasticity, and mesh size at the molecular level of the pNP by controlling the PEG/ovalbumin (OVA) ratio [207]. The results showed that the OVA pNPs led to a significant increase in median survival relative to solute OVA antigens in a B16F10-OVA melanoma mouse model. In addition, Nishit and Si et al. showed that by wrapping cell membranes from different cell sources onto NPs, the active proteins on the cell membranes could endow the NPs with various required functions or adjuvant therapeutic effects, providing a way to enhance cancer immunotherapy [208,209]. Ochyl et al. reported a novel type of PEGylated tumor cell membrane vesicles as a new vaccine platform for tumor immunotherapy, confirmed in a mouse tumor model [210]. The endogenous cell membrane obtained from cancer cells forms PEGylated NPs (PEG-NPs). PEG-NPs show good serum stability in vitro and efficient drainage through local lymph nodes upon subcutaneous administration in vivo. In tumor-bearing mice, treatment with PEG-NPs synthesized by mouse melanoma cells can cause high-efficiency antigen-specific cytotoxic CD8^+^ T lymphocyte responses. Furthermore, Wu et al. reported a surface-layer (S-layer) protein-enhanced immunotherapy strategy based on cell membrane-coated S-CM-HPAD NPs for effective malignant tumor therapy and metastasis inhibition [211]. They proposed that biomimetic S-CM-HPAD NPs have the same targeting, multi-antigen immune activation and drug delivery capabilities, while encapsulated Dox can enhance the immunotherapeutic response and inhibit the growth and metastasis of melanoma tumors by inhibiting myeloid-suppressive cells. It has also been reported that NPs (Natural killer cell NPs, NK-NPs) containing the photosensitizer 4,4′,4″,4‴-(porphine-5,10,15,20-tetrayl) tetrakis (benzoic acid) (TCPP) can eliminate primary tumors and inhibit distant tumors through the NK cell membrane. Deng et al. found that NK-NPs enhanced NK cell membrane immunity through immunogenic photodynamic therapy (PDT) and produced a stronger immune response for tumor-targeted cell membrane immunotherapy [212]. Through proteomic analysis of the NK cell membrane, they proved that the NK cell membrane can target NK-NPs to tumors and initiate the polarization of M1 macrophages to produce cell membrane immunotherapy. Thus, polymer NPs camouflaged by cell membranes have been studied in recent years as powerful drug carriers for improved immunotherapy.

### 3.4. Combination Therapy Based on Polymer Nanocarriers

**Figure 2:**
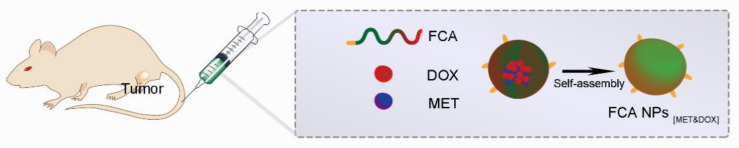
The self-assembled polymeric nanocarrier-mediated co-delivery of metformin and doxorubicin for melanoma therapy Reproduced from Song et al. [111].

Cancer is a complex disease driven by multiple gene mutations, and its progression involves interaction between cancer cells and their microenvironment [213]. Compared to single-agent therapy, combination chemotherapy has shown better clinical treatment effects, especially in delaying the development of cancer chemotherapy resistance [214,215]. Studies have found that cancer cells acquire defense mechanisms by over-expressing drug efflux pumps, increasing drug metabolism, enhancing self-repair capabilities, or expressing altered drug targets, resulting in reduced efficacy and, ultimately, failure of treatment [61]. In order to solve this problem, the use of two or more drugs with different pharmacological mechanisms for combined therapy is a promising treatment strategy (Table 4). For example, in vivo efficacy of dual-drug-loaded NPs was better than that of a single formulation of combretastatin and Dox in mice with B16/F10 melanoma or Lewis lung carcinoma-bearing mice [216]. Currently, various nanodrug delivery systems, such as liposomes and polymer NPs, are used to provide multiple treatments at the same time, including chemotherapeutics, siRNA/mRNA, immunoagonists, photosensitizers, and antiangiogenic agents [217]. In this regard, many polymer NPs have been widely used in the treatment of various cancers. Their advantages are mainly reflected in the prolonged drug half-life, high drug loading rate, low toxicity, controlled release, and specific enrichment. In addition, an important advantage of polymer nanocarriers is that drugs with different physical and chemical properties can be co-encapsulated in the same nanocarrier and delivered to tumor cells simultaneously to achieve combined therapy [218]. In our previous research, we reported the self-assembled polymer nanocarrier-mediated co-delivery of metformin and Dox for the treatment of melanoma [111]. We mainly used folic acid–sodium alginate–cholesteric amphoteric polymer NPs to co-deliver metformin and Dox to melanoma tissues, and to inhibit tumor progression by inducing PANoptosis (pyroptosis, apoptosis, and necroptosis) of melanoma cells (Figure 2). To ensure effective drug release in the target tissue in an ideal manner, several strategies have been developed involving introducing stimulus responsiveness into polymer NPs, giving them a specific ability to change their structure or chemical composition in response to slight changes in the environment, which then triggers drug release. Guo et al. designed a dual-pH responsive biopolymer–Dox conjugate NP to encapsulate lapatinib [219]. In an acidic tumor environment, the surface charge conversion of NPs can be triggered, and the uptake of drug-loaded NPs and the release of drugs can be promoted by tumor cells. This is expected to have an improved therapeutic effect on breast cancer. In addition to pH-sensitive nanocarriers, Liu and his collaborators successfully prepared and modified glutathione (GSH)-responsive Fe-DSCP NPs constructed from Fe3^+^ and the cisplatin prodrug (DSCP) [220]. This cRGD-conjugated Fe-DSCP-PEG NP (Fe-DSCP-PEG–cRGD) can be used as a tumor-specific therapeutic drug for the combined therapy of targeted chemotherapy and chemotherapy kinetics. In addition, researchers have found that biomimetic NPs have great potential in combination therapy. Wang et al. proposed that the use of erythrocyte cell membranes to disguise NPs could efficiently deliver photosensitizers and pro-hypoxic drugs in combined tumor therapy [221]. Similarly, Cong Xu et al. developed a biomimetic dual-drug delivery system (Si/PNPs@HeLa) that simultaneously targeted the delivery of PTX and siRNA by camouflaging HeLa cell membranes onto siRNA/PTX co-loaded PLGA NPs [222]. In summary, we have reason to believe that combined therapy mediated by nanodrug delivery systems will bring about a new dawn for the personalized treatment of tumors.

## 4. Conclusions

Recent studies on NDDSs have shown that, compared to traditional chemotherapy or immunotherapy, these systems have great advantages in targeted drug delivery. Moreover, the unique physical and chemical properties of NP drug carriers make them very suitable for tumor therapy [86,227]. However, despite many efforts in the development of new targeted nanocarriers (including organic polymers and mixed systems with inorganic materials such as gold, silver, and silicon oxide), only a few nanocarriers have been approved for clinical use [228]. This phenomenon may be due to the lack of non-specific distribution and the accumulation of targeted NPs after administration, and concerns about their safety. In order to fully evaluate the advantages and disadvantages of NP therapy, more clinical data are needed, which also helps to optimize the development of nanomedicines. In this paper, we summarized the application of polymer nanodrug delivery systems in tumor treatment. Compared to other systems, this type of drug delivery system contains highlights such as its unique high drug loading efficiency and targeting ability. Safety issues are key to designing an optimal NDDS. We found that different polymer NPs have been proven to be safe in many studies, indicating that polymer NP carriers may be a potential ideal drug delivery carrier for the clinical treatment of tumors.

With the development of NDDSs, advances in nanophototherapy/early diagnosis technology have indicated that there is development potential for multi-functional “smart” NPs, which may help to achieve individualized cancer treatment [229]. Especially in cancer treatment, early detection of growing tumor cells is crucial, and determines the success or failure of the treatment [230]. Nano imaging agent-based fluorescence imaging is an easy diagnostic technique which provides high spatial and temporal resolution, excellent sensitivity and good selectivity [231,232]. Therefore, without the need for anatomic intervention, such as the use of an endoscope or a microfiber catheter, fluorescence imaging provides the ability to diagnose the cancer organism with high sensitivity. For the practical application of fluorescence nanotechnology (FNP) in the practice of in vivo surgery, the following requirements must be followed: (i) FNP should be demonstrated with high purity and non-toxicity to ensure safe management, (ii) the biological system should have excellent colloidal stability to avoid degradation or aggregation and to increase blood circulation time, (iii) complete removal from the biological system should be guaranteed after the imaging process is completed, and (iv) dyes containing NPs should have high stable fluorescence to ensure long-term imaging and a good signal-to-noise ratio [233]. Previously, we emphasized that polymer NPs have excellent biocompatibility, low toxicity or non-toxicity, and can form long-term stable particles in the biological environment. Meanwhile, polymer NPs can be loaded with fluorescein multiple times to cause fluorescence enhancement, protect the dye in the nanoparticle core from the biological environment, and avoid unnecessary side effects, such as decreased fluorescence caused by protein interactions [234,235]. Continuous research on NPs in preclinical and clinical research will improve the prevention, diagnosis, and treatment of cancer.

## Figures and Tables

**Table 1:** List of polymer NPs for Cancer Chemotherapy.

Drug Name	Type of Nanocarriers	Cancer Type	Reference
Taxol (PTX)	Self-assembled lipid NPs	Breast, ovary, and lung	[145]
Folex (Methotrexate)	Lipid-polymer hybrid NPs	Breast, lung, blood, bone, and lymph system	[146]
Adriamycin (Dox)	PLGA NPs and chitosan NPs	Breast cancer, lymphoma, and multiple myeloma	[147,148]
Platinol-AQ (Cisplatin)	ScFvEGFR-heparin-cisplatin;	Bladder, ovary, lung, and testicles	[149,150,151,152]
Oncovin (Vincristine)	Peptide R7-conjugated PLGA-PEG NPs;	Leukemia and lymphoma	[153]
5-FU (Fluorouracil)	Chitosan NPs and solid lipid NPs;	Colon, breast, stomach, and head and neck	[154,155]
Gemzar (Gemcitabine)	Polyketal NPs and lipid polymer hybrid;	Pancreas, breast, ovary, and lung	[156,157]

**Table 2:** List of polymer NPs in gene therapy for cancer treatment.

Gene	NPs (Class)	Tumor Type	Reference
mRNA (P53)	Paclitaxel amino lipid NPs	Breast cancer	[184]
siRNA (CDK1)	Aptamer-protamine-siRNA NPs and Carboxylated graphene oxide-trimethyl chitosan-hyaluronate NPs	Breast cancer, melanoma, and colorectal cancer	[185,186]
CRISPR/Cas9 genome editing	Phenylboronic acid-derived lipid NPs	Cervical cancer	[187]
miRNA (c-Myc)	polymeric CXCR4 antagonists NPs	Human malignant cholangiocarcinoma	[188]

**Table 3:** NPs-based immunotherapy for cancer treatment.

Drug Name	Class of Treatment	Tumor Type	Reference
Keytruda (Pembrolizumab)	Checkpoint Inhibitor (PD-1 inhibitor)	Melanoma and non–small cell lung cancer	[200,201]
Yervoy (Ipilimumab)	Checkpoint Inhibitor (CTLA-4 inhibitor)	Melanoma	[202,203]
Imfinzi (Durvalumab)	Checkpoint Inhibitor (PD-L1 inhibitor)	Non-small cell lung cancer	[204]
Kymriah (Tisagenlecleucel)	CAR T-cell therapy	Large B-cell lymphoma	[205]
Provenge (Sipuleucel-T)	Cancer Vaccines	Prostate cancer	[206]

**Table 4:** List of polymer NPs for combination therapy.

Therapeutic Agents	NPs	Tumor Type	Reference
Paclitaxel/lonidamine	Polymeric PLGA-PEG NPs, PCL NPs	Breast cancer	[223]
DOX/combretastatin	Lipid-polymer hybrid NPs, PLGA NPs	Lung	[216,224]
DOX/metformin	Folic acid-cholesterolsodium alginate NPs	Melanoma	[111]
PTX/siRNA	layer-by-layer NPs	Lung	[225]
SiRNA/OVA	PLGA NPs	Melanoma	[226]

## References

[1] Reyes-Farias M., Carrasco-Pozo C. (2019). The anti-cancer effect of quercetin: Molecular implications in cancer metabolism. Int. J. Mol. Sci..

[2] Cortes J., Perez-García J.M., Llombart-Cussac A., Curigliano G., El Saghir N.S., Cardoso F., Barrios C.H., Wagle S., Roman J., Harbeck N. (2020). Enhancing global access to cancer medicines. CA Cancer J. Clin..

[3] Li Y., Liu W., Zhao L., Güngör C., Xu Y., Song X., Wang D., Zhou Z., Zhou Y., Li C. (2020). Nomograms predicting overall survival and cancer-specific survival for synchronous colorectal liver-limited metastasis. J. Cancer Res. Clin. Oncol..

[4] Wang N., Mengersen K., Tong S., Kimlin M., Zhou M., Hu W. (2020). Global, regional, and national burden of lung cancer and its attributable risk factors, 1990 to 2017. Cancer.

[5] Sung H., Ferlay J., Siegel R.L., Laversanne M., Soerjomataram I., Jemal A., Bray F. (2021). Global cancer statistics 2020: GLOBOCAN estimates of incidence and mortality worldwide for 36 cancers in 185 countries. CA Cancer J Clin.

[6] Ferlay J., Colombet M., Soerjomataram I., Parkin D.M., Piñeros M., Znaor A., Bray F. (2021). Cancer statistics for the year 2020: An overview. Int. J. Cancer.

[7] Dhillon P.K., Mathur P., Nandakumar A., Fitzmaurice C., Kumar G.A., Mehrotra R., Shukla D., Rath G., Gupta P.C., Swaminathan R. (2018). The burden of cancers and their variations across the states of India: The Global Burden of Disease Study 1990–2016. Lancet Oncol..

[8] Bailar J.C., Gornik H.L. (1997). Cancer undefeated. N. Engl J. Med..

[9] Sullivan R., Alatise O.I., Anderson B.O., Audisio R., Autier P., Aggarwal A., Balch C., Brennan M.F., Dare A., D’Cruz A. (2015). Global cancer surgery: Delivering safe, affordable, and timely cancer surgery. Lancet Oncol..

[10] Strobel O., Hank T., Hinz U., Bergmann F., Schneider L., Springfeld C., Jäger D., Schirmacher P., Hackert T., Büchler M.W. (2017). Pancreatic cancer surgery. Ann. Surg..

[11] Rudra S., Jiang N., Rosenberg S.A., Olsen J.R., Roach M.C., Wan L., Portelance L., Mellon E.A., Bruynzeel A., Lagerwaard F. (2019). Using adaptive magnetic resonance image-guided radiation therapy for treatment of inoperable pancreatic cancer. Cancer Med..

[12] de Crevoisier R., Bayar M.A., Pommier P., Muracciole X., Pêne F., Dudouet P., Latorzeff I., Beckendorf V., Bachaud J.-M., Laplanche A. (2018). Daily versus weekly prostate cancer image guided radiation therapy: Phase 3 multicenter randomized trial. Int. J. Radiat. Oncol. Biol. Phys..

[13] Deng J., Xu S., Hu W., Xun X., Zheng L., Su M. (2018). Tumor targeted, stealthy and degradable bismuth nanoparticles for enhanced X-ray radiation therapy of breast cancer. Biomaterials.

[14] DeVita V.T., Chu E. (2008). A history of cancer chemotherapy. Cancer Res..

[15] Yang H., Villani R.M., Wang H., Simpson M.J., Roberts M.S., Tang M., Liang X. (2018). The role of cellular reactive oxygen species in cancer chemotherapy. J. Exp. Clin. Cancer Res..

[16] Bukowski K., Kciuk M., Kontek R. (2020). Mechanisms of multidrug resistance in cancer chemotherapy. Int. J. Mol. Sci..

[17] Shi T., Zhu J., Feng Y., Tu D., Zhang Y., Zhang P., Jia H., Huang X., Cai Y., Yin S. (2021). Secondary cytoreduction followed by chemotherapy versus chemotherapy alone in platinum-sensitive relapsed ovarian cancer (SOC-1): A multicentre, open-label, randomised, phase 3 trial. Lancet Oncol..

[18] Ye Z., Huang Y., Ke J., Zhu X., Leng S., Luo H. (2021). Breakthrough in targeted therapy for non-small cell lung cancer. Biomed. Pharmacother..

[19] Paris A., Tardif N., Galibert M.-D., Corre S. (2021). AhR and cancer: From gene profiling to targeted therapy. Int. J. Mol. Sci..

[20] Fox G.C., Su X., Davis J.L., Xu Y., Kwakwa K.A., Ross M.H., Fontana F., Xiang J., Esser A.K., Cordell E. (2021). Targeted therapy to β3 integrin reduces chemoresistance in breast cancer bone metastases. Mol. Cancer Ther..

[21] Wilkinson S., Ye H., Karzai F., Harmon S.A., Terrigino N.T., VanderWeele D.J., Bright J.R., Atway R., Trostel S.Y., Carrabba N.V. (2021). Nascent prostate cancer heterogeneity drives evolution and resistance to intense hormonal therapy. Eur. Urol..

[22] Liu J.M., Lin C.C., Chen M.F., Liu K.L., Lin C.F., Chen T.H., Wu C.T. (2021). Risk of major adverse cardiovascular events among second-line hormonal therapy for metastatic castration-resistant prostate cancer: A real-world evidence study. Prostate.

[23] Gong N., Sheppard N.C., Billingsley M.M., June C.H., Mitchell M.J. (2021). Nanomaterials for T-cell cancer immunotherapy. Nat. Nanotechnol..

[24] Liu S., Jiang Q., Zhao X., Zhao R., Wang Y., Wang Y., Liu J., Shang Y., Zhao S., Wu T. (2021). A DNA nanodevice-based vaccine for cancer immunotherapy. Nat. Mater..

[25] Sharifi M., Jafari S., Hasan A., Paray B.A., Gong G., Zheng Y., Falahati M. (2020). Antimetastatic activity of lactoferrin-coated mesoporous maghemite nanoparticles in breast cancer enabled by combination therapy. ACS Biomater. Sci. Eng..

[26] Levit S.L., Tang C. (2021). Polymeric Nanoparticle Delivery of Combination Therapy with Synergistic Effects in Ovarian Cancer. Nanomaterials.

[27] Shi C., Zhou Z., Lin H., Gao J. (2021). Imaging Beyond Seeing: Early Prognosis of Cancer Treatment. Small Methods.

[28] Herrmann J. (2020). Adverse cardiac effects of cancer therapies: Cardiotoxicity and arrhythmia. Nat. Rev. Cardiol..

[29] Zhang C.-L., Huang T., Wu B.-L., He W.-X., Liu D. (2017). Stem cells in cancer therapy: Opportunities and challenges. Oncotarget.

[30] Gupta G.P., Massagué J. (2006). Cancer metastasis: Building a framework. Cell.

[31] Mahvi D.A., Liu R., Grinstaff M.W., Colson Y.L., Raut C.P. (2018). Local cancer recurrence: The realities, challenges, and opportunities for new therapies. CA Cancer J. Clin..

[32] Turajlic S., Sottoriva A., Graham T., Swanton C. (2019). Resolving genetic heterogeneity in cancer. Nat. Rev. Genet..

[33] Hu J., Wang W., Lan X., Zeng Z., Liang Y., Yan Y., Song F., Wang F., Zhu X., Liao W. (2019). CAFs secreted exosomes promote metastasis and chemotherapy resistance by enhancing cell stemness and epithelial-mesenchymal transition in colorectal cancer. Mol. Cancer.

[34] Gobin E., Bagwell K., Wagner J., Mysona D., Sandirasegarane S., Smith N., Bai S., Sharma A., Schleifer R., She J.-X. (2019). A pan-cancer perspective of matrix metalloproteases (MMP) gene expression profile and their diagnostic/prognostic potential. BMC Cancer.

[35] Annovazzi L., Mellai M., Schiffer D. (2017). Chemotherapeutic drugs: DNA damage and repair in glioblastoma. Cancers.

[36] Muhamad N., Plengsuriyakarn T., Na-Bangchang K. (2018). Application of active targeting nanoparticle delivery system for chemotherapeutic drugs and traditional/herbal medicines in cancer therapy: A systematic review. Int. J. Nanomed..

[37] Pich O., Muiños F., Lolkema M.P., Steeghs N., Gonzalez-Perez A., Lopez-Bigas N. (2019). The mutational footprints of cancer therapies. Nat. Genet..

[38] Maeda H., Khatami M. (2018). Analyses of repeated failures in cancer therapy for solid tumors: Poor tumor-selective drug delivery, low therapeutic efficacy and unsustainable costs. Clin. Transl. Med..

[39] Zheng H., Bae Y., Kasimir-Bauer S., Tang R., Chen J., Ren G., Yuan M., Esposito M., Li W., Wei Y. (2017). Therapeutic antibody targeting tumor-and osteoblastic niche-derived Jagged1 sensitizes bone metastasis to chemotherapy. Cancer Cell.

[40] Larionova I., Cherdyntseva N., Liu T., Patysheva M., Rakina M., Kzhyshkowska J. (2019). Interaction of tumor-associated macrophages and cancer chemotherapy. Oncoimmunology.

[41] Karagiannis G.S., Condeelis J.S., Oktay M.H. (2019). Chemotherapy-induced metastasis: Molecular mechanisms, clinical manifestations, therapeutic interventions. Cancer Res..

[42] Lankelma J., Dekker H., Luque R.F., Luykx S., Hoekman K., Van Der Valk P., Van Diest P.J., Pinedo H.M. (1999). Doxorubicin gradients in human breast cancer. Clin. Cancer. Res..

[43] Bollag G., Tsai J., Zhang J., Zhang C., Ibrahim P., Nolop K., Hirth P. (2012). Vemurafenib: The first drug approved for BRAF-mutant cancer. Nat. Rev. Drug Discov..

[44] Markman M., Mekhail T.M. (2002). Paclitaxel in cancer therapy. Expert Opin. Pharm..

[45] Patel A.G., Kaufmann S.H. (2012). Cancer: How does doxorubicin work?. Elife.

[46] Lind M. (2008). Principles of cytotoxic chemotherapy. Medicine.

[47] Xie Y., Chen Y., Fang J. (2020). Comprehensive review of targeted therapy for colorectal cancer. Signal Transduct. Target. Ther..

[48] Ayati A., Moghimi S., Salarinejad S., Safavi M., Pouramiri B., Foroumadi A. (2020). A review on progression of epidermal growth factor receptor (EGFR) inhibitors as an efficient approach in cancer targeted therapy. Bioorg. Chem..

[49] Hegde P.S., Chen D.S. (2020). Top 10 challenges in cancer immunotherapy. Immunity.

[50] Waldman A.D., Fritz J.M., Lenardo M.J. (2020). A guide to cancer immunotherapy: From T cell basic science to clinical practice. Nat. Rev. Immunol..

[51] Tan S., Li D., Zhu X. (2020). Cancer immunotherapy: Pros, cons and beyond. Biomed. Pharmacother..

[52] He X., Xu C. (2020). Immune checkpoint signaling and cancer immunotherapy. Cell Res..

[53] Kennedy L.B., Salama A.K. (2020). A review of cancer immunotherapy toxicity. CA Cancer J. Clin..

[54] Nikolaou M., Pavlopoulou A., Georgakilas A.G., Kyrodimos E. (2018). The challenge of drug resistance in cancer treatment: A current overview. Clin. Exp. Metastasis.

[55] Norouzi-Barough L., Sarookhani M.R., Sharifi M., Moghbelinejad S., Jangjoo S., Salehi R. (2018). Molecular mechanisms of drug resistance in ovarian cancer. J. Cell. Physiol..

[56] Zahreddine H., Borden K. (2013). Mechanisms and insights into drug resistance in cancer. Front. Pharmacol..

[57] Stavrovskaya A. (2000). Cellular mechanisms of multidrug resistance of tumor cells. Biochem. C/C Biokhimiia.

[58] Du Y.-Z., Wang L., Yuan H., Hu F.-Q. (2011). Linoleic acid-grafted chitosan oligosaccharide micelles for intracellular drug delivery and reverse drug resistance of tumor cells. Int. J. Biol. Macromol..

[59] Lu C., Guan J., Lu S., Jin Q., Rousseau B., Lu T., Stephens D., Zhang H., Zhu J., Yang M. (2021). DNA sensing in mismatch repair-deficient tumor cells is essential for anti-tumor immunity. Cancer Cell.

[60] Peetla C., Vijayaraghavalu S., Labhasetwar V. (2013). Biophysics of cell membrane lipids in cancer drug resistance: Implications for drug transport and drug delivery with nanoparticles. Adv. Drug Del. Rev..

[61] Gottesman M.M. (2002). Mechanisms of cancer drug resistance. Annu. Rev. Med..

[62] Rini B.I. (2010). New strategies in kidney cancer: Therapeutic advances through understanding the molecular basis of response and resistance. Clin. Cancer. Res..

[63] Wei L., Wang X., Lv L., Liu J., Xing H., Song Y., Xie M., Lei T., Zhang N., Yang M. (2019). The emerging role of microRNAs and long noncoding RNAs in drug resistance of hepatocellular carcinoma. Mol. Cancer.

[64] Helmbach H., Rossmann E., Kern M.A., Schadendorf D. (2001). Drug-resistance in human melanoma. Int. J. Cancer.

[65] Sun H., Su J., Meng Q., Yin Q., Chen L., Gu W., Zhang P., Zhang Z., Yu H., Wang S. (2016). Cancer-cell-biomimetic nanoparticles for targeted therapy of homotypic tumors. Adv. Mater..

[66] Xu S., Wang L., Liu Z. (2021). Molecularly imprinted polymer nanoparticles: An emerging versatile platform for cancer therapy. Angew. Chem. Int. Ed..

[67] Zhao W., Li T., Long Y., Guo R., Sheng Q., Lu Z., Li M., Li J., Zang S., Zhang Z.J.A.A.M. (2021). Self-promoted Albumin-Based Nanoparticles for Combination Therapy against Metastatic Breast Cancer via a Hyperthermia-Induced “Platelet Bridge”. ACS Appl. Mater. Interfaces.

[68] Kim J., Shim M.K., Yang S., Moon Y., Song S., Choi J., Kim J., Kim K. (2021). Combination of cancer-specific prodrug nanoparticle with Bcl-2 inhibitor to overcome acquired drug resistance. J. Control. Release.

[69] Chen D., Wu Y., Hoque S., Tilley R.D., Gooding J.J. (2021). Rapid and ultrasensitive electrochemical detection of circulating tumor DNA by hybridization on the network of gold-coated magnetic nanoparticles. Chem. Sci..

[70] Jain P., Kathuria H., Momin M. (2021). Clinical therapies and nano drug delivery systems for urinary bladder cancer. Pharmacol. Ther..

[71] Zhang X., Liang T., Ma Q. (2021). Layer-by-Layer assembled nano-drug delivery systems for cancer treatment. Drug Deliv..

[72] Karaman D.Ş., Kaasalainen M., Kettiger H., Rosenholm J.M. (2021). Opportunities and Challenges of Silicon-based Nanoparticles for Drug Delivery and Imaging. Charact. Pharm. Nano Microsyst..

[73] Sponchioni M., Palmiero U.C., Moscatelli D. (2019). Thermo-responsive polymers: Applications of smart materials in drug delivery and tissue engineering. Mater. Sci. Eng. C.

[74] Prasher P., Sharma M., Singh S.P. (2021). Drug encapsulating polysaccharide-loaded metal nanoparticles: A perspective drug delivery system. Drug Dev. Res..

[75] Wichaita W., Kim Y.-G., Tangboriboonrat P., Thérien-Aubin H. (2020). Polymer-functionalized polymer nanoparticles and their behaviour in suspensions. Polym. Chem..

[76] Lu H., Yang G., Ran F., Gao T., Sun C., Zhao Q., Wang S. (2020). Polymer-functionalized mesoporous carbon nanoparticles on overcoming multiple barriers and improving oral bioavailability of Probucol. Carbohydr. Polym..

[77] Zielińska A., Skwarek E., Zaleska A., Gazda M., Hupka J. (2009). Preparation of silver nanoparticles with controlled particle size. Procedia Chem..

[78] Garcês A., Amaral M., Lobo J.S., Silva A.C. (2018). Formulations based on solid lipid nanoparticles (SLN) and nanostructured lipid carriers (NLC) for cutaneous use: A review. Eur. J. Pharm. Sci..

[79] Yang Y., He J., Li Q., Gao L., Hu J., Zeng R., Qin J., Wang S.X., Wang Q. (2019). Self-healing of electrical damage in polymers using superparamagnetic nanoparticles. Nat. Nanotechnol..

[80] Yang G., Phua S.Z.F., Bindra A.K., Zhao Y. (2019). Degradability and clearance of inorganic nanoparticles for biomedical applications. Adv. Mater..

[81] He C., Cheng J., Zhang X., Douthwaite M., Pattisson S., Hao Z. (2019). Recent advances in the catalytic oxidation of volatile organic compounds: A review based on pollutant sorts and sources. Chem. Rev..

[82] Jamkhande P.G., Ghule N.W., Bamer A.H., Kalaskar M.G. (2019). Metal nanoparticles synthesis: An overview on methods of preparation, advantages and disadvantages, and applications. J. Drug Deliv. Sci. Technol..

[83] Glassman P.M., Muzykantov V.R.J.J.o.P., Therapeutics E. (2019). Pharmacokinetic and pharmacodynamic properties of drug delivery systems. J. Pharmacol. Exp. Ther..

[84] Chouaib R., Sarieddine R., Gali-Muhtasib H. (2020). Nanoparticles as Drug Delivery Systems for Cancer Treatment: Applications in Targeted Therapy and Personalized Medicine. Nanopart. Drug Deliv. Syst. Cancer Treat..

[85] Dudhipala N., Gorre T. (2020). Neuroprotective effect of ropinirole lipid nanoparticles enriched hydrogel for parkinson’s disease: In vitro, ex vivo, pharmacokinetic and pharmacodynamic evaluation. Pharmaceutics.

[86] Sukhanova A., Bozrova S., Sokolov P., Berestovoy M., Karaulov A., Nabiev I. (2018). Dependence of nanoparticle toxicity on their physical and chemical properties. Nanoscale Res. Lett..

[87] Xu C., Qiao L., Guo Y., Ma L., Cheng Y. (2018). Preparation, characteristics and antioxidant activity of polysaccharides and proteins-capped selenium nanoparticles synthesized by Lactobacillus casei ATCC 393. Carbohydr. Polym..

[88] Kister T., Monego D., Mulvaney P., Widmer-Cooper A., Kraus T. (2018). Colloidal stability of apolar nanoparticles: The role of particle size and ligand shell structure. ACS Nano.

[89] Quinson J., Inaba M., Neumann S., Swane A.A., Bucher J., Simonsen S.B., Theil Kuhn L., Kirkensgaard J.J., Jensen K.M., Oezaslan M. (2018). Investigating particle size effects in catalysis by applying a size-controlled and surfactant-free synthesis of colloidal nanoparticles in alkaline ethylene glycol: Case study of the oxygen reduction reaction on Pt. ACS Catal..

[90] Caster J.M., Stephanie K.Y., Patel A.N., Newman N.J., Lee Z.J., Warner S.B., Wagner K.T., Roche K.C., Tian X., Min Y. (2017). Effect of particle size on the biodistribution, toxicity, and efficacy of drug-loaded polymeric nanoparticles in chemoradiotherapy. Nanomed. Nanotechnol. Biol. Med..

[91] Huang T., Holden J.A., Heath D.E., O’Brien-Simpson N.M., O’Connor A.J. (2019). Engineering highly effective antimicrobial selenium nanoparticles through control of particle size. Nanoscale.

[92] Liu S., Yuen M.C., White E.L., Boley J.W., Deng B., Cheng G.J., Kramer-Bottiglio R. (2018). Laser sintering of liquid metal nanoparticles for scalable manufacturing of soft and flexible electronics. ACS Appl. Mater. Interfaces.

[93] Grodzinski P., Silver M., Molnar L.K. (2006). Nanotechnology for cancer diagnostics: Promises and challenges. Expert Rev. Mol. Diagn..

[94] Li S.-D., Huang L. (2008). Pharmacokinetics and biodistribution of nanoparticles. Mol. Pharm..

[95] Xiao W., Gao H. (2018). The impact of protein corona on the behavior and targeting capability of nanoparticle-based delivery system. Int. J. Pharm..

[96] Anchordoquy T.J., Barenholz Y., Boraschi D., Chorny M., Decuzzi P., Dobrovolskaia M.A., Farhangrazi Z.S., Farrell D., Gabizon A., Ghandehari H. (2017). Mechanisms and Barriers in Cancer Nanomedicine: Addressing Challenges, Looking for Solutions. ACS Nano.

[97] Petros R.A., DeSimone J.M. (2010). Strategies in the design of nanoparticles for therapeutic applications. Nat. Rev. Drug Discov..

[98] Rastgar M., Shakeri A., Bozorg A., Salehi H., Saadattalab V. (2017). Impact of nanoparticles surface characteristics on pore structure and performance of forward osmosis membranes. Desalination.

[99] Hou L., Liang Q., Wang F. (2020). Mechanisms that control the adsorption–desorption behavior of phosphate on magnetite nanoparticles: The role of particle size and surface chemistry characteristics. RSC Adv..

[100] Settanni G., Zhou J., Suo T., Schöttler S., Landfester K., Schmid F., Mailänder V. (2017). Protein corona composition of poly (ethylene glycol)-and poly (phosphoester)-coated nanoparticles correlates strongly with the amino acid composition of the protein surface. Nanoscale.

[101] Partikel K., Korte R., Stein N.C., Mulac D., Herrmann F.C., Humpf H.-U., Langer K. (2019). Effect of nanoparticle size and PEGylation on the protein corona of PLGA nanoparticles. Eur. J. Pharm. Biopharm..

[102] Yang X., Hu C., Tong F., Liu R., Zhou Y., Qin L., Ouyang L., Gao H. (2019). Tumor Microenvironment-Responsive Dual Drug Dimer-Loaded PEGylated Bilirubin Nanoparticles for Improved Drug Delivery and Enhanced Immune-Chemotherapy of Breast Cancer. Adv. Funct. Mater..

[103] Xiao W., Xiong J., Zhang S., Xiong Y., Zhang H., Gao H. (2018). Influence of ligands property and particle size of gold nanoparticles on the protein adsorption and corresponding targeting ability. Int. J. Pharm..

[104] Patsula V., Horák D., Kučka J., Macková H., Lobaz V., Francová P., Herynek V., Heizer T., Páral P., Šefc L. (2019). Synthesis and modification of uniform PEG-neridronate-modified magnetic nanoparticles determines prolonged blood circulation and biodistribution in a mouse preclinical model. Sci. Rep..

[105] Riley T., Stolnik S., Heald C., Xiong C., Garnett M., Illum L., Davis S., Purkiss S., Barlow R., Gellert P. (2001). Physicochemical evaluation of nanoparticles assembled from Poly (lactic acid)−Poly (ethylene glycol)(PLA−PEG) block copolymers as drug delivery vehicles. Langmuir.

[106] Chen H.-Y., Deng J., Wang Y., Wu C.-Q., Li X., Dai H.-W. (2020). Hybrid cell membrane-coated nanoparticles: A multifunctional biomimetic platform for cancer diagnosis and therapy. Acta Biomater..

[107] Hu C.-M.J., Zhang L., Aryal S., Cheung C., Fang R.H., Zhang L. (2011). Erythrocyte membrane-camouflaged polymeric nanoparticles as a biomimetic delivery platform. Proc. Natl. Acad. Sci. USA.

[108] Blanco E., Shen H., Ferrari M. (2015). Principles of nanoparticle design for overcoming biological barriers to drug delivery. Nat. Biotechnol..

[109] Lin G., Zhang H., Huang L. (2015). Smart polymeric nanoparticles for cancer gene delivery. Mol. Pharm..

[110] Zhang M., Guo X., Wang M., Liu K. (2020). Tumor microenvironment-induced structure changing drug/gene delivery system for overcoming delivery-associated challenges. J. Control. Release.

[111] Song M., Xia W., Tao Z., Zhu B., Zhang W., Liu C., Chen S. (2021). Self-assembled polymeric nanocarrier-mediated co-delivery of metformin and doxorubicin for melanoma therapy. Drug Deliv..

[112] Chen S., Zhang W., Sun C., Song M., Liu S., Xu M., Zhang X., Liu L., Liu C. (2020). Systemic Nanoparticle-Mediated Delivery of Pantetheinase Vanin-1 Regulates Lipolysis and Adiposity in Abdominal White Adipose Tissue. Adv. Sci. (Weinh).

[113] Nguyen K.T. (2011). Targeted Nanoparticles for Cancer Therapy: Promises and Challenges. J. Nanomed. Nanotechnol..

[114] Wang X., Xu J., Xu X., Fang Q., Tang R. (2020). pH-sensitive bromelain nanoparticles by ortho ester crosslinkage for enhanced doxorubicin penetration in solid tumor. Mater. Sci. Eng. C Mater. Biol. Appl..

[115] Li Y., Cong H., Wang S., Yu B., Shen Y. (2020). Liposomes modified with bio-substances for cancer treatment. Biomater. Sci..

[116] Chiang Y.-T., Lo C.-L. (2014). pH-responsive polymer-liposomes for intracellular drug delivery and tumor extracellular matrix switched-on targeted cancer therapy. Biomaterials.

[117] Ooi Y.J., Wen Y., Zhu J., Song X., Li J. (2020). Surface charge switchable polymer/DNA nanoparticles responsive to tumor extracellular ph for tumor-triggered enhanced gene delivery. Biomacromolecules.

[118] Chen C.-K., Huang P.-K., Law W.-C., Chu C.-H., Chen N.-T., Lo L.-W. (2020). Biodegradable polymers for gene-delivery applications. Int. J. Nanomed..

[119] Liao Z., Wong S.W., Yeo H.L., Zhao Y. (2020). Nanocarriers for cancer treatment: Clinical impact and safety. NanoImpact.

[120] Wolfram J., Zhu M., Yang Y., Shen J., Gentile E., Paolino D., Fresta M., Nie G., Chen C., Shen H. (2015). Safety of nanoparticles in medicine. Curr. Drug Targets.

[121] Mocan T., Clichici S., Agoşton-Coldea L., Mocan L., Şimon Ş., Ilie I., Biriş A., Mureşan A. (2010). Implications of oxidative stress mechanisms in toxicity of nanoparticles. Acta Physiol. Hung..

[122] Taguchi K., Lu H., Jiang Y., Hung T.T., Stenzel M.H. (2018). Safety of nanoparticles based on albumin–polymer conjugates as a carrier of nucleotides for pancreatic cancer therapy. J. Mater. Chem. B.

[123] Hickey J.W., Santos J.L., Williford J.-M., Mao H.-Q. (2015). Control of polymeric nanoparticle size to improve therapeutic delivery. J. Control. Release.

[124] Zielinska A., Carreiro F., Oliveira A.M., Neves A., Pires B., Venkatesh D.N., Durazzo A., Lucarini M., Eder P., Silva A.M. (2020). Polymeric Nanoparticles: Production, Characterization, Toxicology and Ecotoxicology. Molecules.

[125] Lima T., Bernfur K., Vilanova M., Cedervall T. (2020). Understanding the lipid and protein corona formation on different sized polymeric nanoparticles. Sci. Rep..

[126] Liu Y., Yang G., Baby T., Chen D., Weitz D.A., Zhao C.X. (2020). Stable polymer nanoparticles with exceptionally high drug loading by sequential nanoprecipitation. Angew. Chem..

[127] Chen J., Qi J., Chen C., Chen J., Liu L., Gao R., Zhang T., Song L., Ding D., Zhang P. (2020). Tocilizumab–Conjugated Polymer Nanoparticles for NIR-II Photoacoustic-Imaging-Guided Therapy of Rheumatoid Arthritis. Adv. Mater..

[128] Liu C., Lu D., You X., Shi G., Deng J., Zhou T. (2020). Carbon dots sensitized lanthanide infinite coordination polymer nanoparticles: Towards ratiometric fluorescent sensing of cerebrospinal Aβ monomer as a biomarker for Alzheimer’s disease. Anal. Chim. Acta.

[129] Song M., Li L., Zhang Y., Chen K., Wang H., Gong R. (2017). Carboxymethyl-β-cyclodextrin grafted chitosan nanoparticles as oral delivery carrier of protein drugs. React. Funct. Polym..

[130] Jiang Y., Liu B., Xu J., Pan K., Hou H., Hu J., Yang J. (2018). Cross-linked chitosan/β-cyclodextrin composite for selective removal of methyl orange: Adsorption performance and mechanism. Carbohydr. Polym..

[131] Dhar S., Gu F.X., Langer R., Farokhzad O.C., Lippard S.J. (2008). Targeted delivery of cisplatin to prostate cancer cells by aptamer functionalized Pt (IV) prodrug-PLGA–PEG nanoparticles. Proc. Natl. Acad. Sci. USA.

[132] Yang J., Kopeček J. (2017). The light at the end of the tunnel—Second generation HPMA conjugates for cancer treatment. Curr. Opin. Colloid Interface Sci..

[133] Muss H.B., Cooper M.R., Brockschmidt J.K., Ferree C., Richards F., White D.R., Jackson D.V., Spurr C.L. (1991). A randomized trial of chemotherapy (L-PAM vs CMF) and irradiation for node positive breast cancer. Breast Cancer Res. Treat..

[134] Bordat A., Boissenot T., Nicolas J., Tsapis N. (2019). Thermoresponsive polymer nanocarriers for biomedical applications. Adv. Drug Del. Rev..

[135] Hyun H., Park J., Willis K., Park J.E., Lyle L.T., Lee W., Yeo Y. (2018). Surface modification of polymer nanoparticles with native albumin for enhancing drug delivery to solid tumors. Biomaterials.

[136] Subjakova V., Oravczova V., Hianik T. (2021). Polymer Nanoparticles and Nanomotors Modified by DNA/RNA Aptamers and Antibodies in Targeted Therapy of Cancer. Polymers.

[137] Song M., Liu C., Chen S., Zhang W. (2021). Nanocarrier-Based Drug Delivery for Melanoma Therapeutics. Int. J. Mol. Sci..

[138] Fu D., Calvo J.A., Samson L.D. (2012). Balancing repair and tolerance of DNA damage caused by alkylating agents. Nat. Rev. Cancer.

[139] Perez R.L., Münz F., Vidoni D., Rühle A., Trinh T., Sisombath S., Zou B., Wuchter P., Debus J., Grosu A.-L. (2019). Mesenchymal stem cells preserve their stem cell traits after exposure to antimetabolite chemotherapy. Stem Cell Res..

[140] Tenconi E., Rigali S. (2018). Self-resistance mechanisms to DNA-damaging antitumor antibiotics in actinobacteria. Curr. Opin. Microbiol..

[141] Shi J.-f., Li J., Yang X.-q., Zhang Y., Yang S.-c., Luo Y.-y., Zhang J.-m., Fu C.-m. (2019). Antitumor status analysis on the co-delivery systems regarding the active ingredients of Chinese herbs combined with chemotherapeutic drugs. Acta Pharm. Sin..

[142] Lissoni P., Rovelli F., Brivio F., Messina G., Lissoni A., Pensato S., Di Fede G. (2018). Five year-survivals with high-dose melatonin and other antitumor pineal hormones in advanced cancer patients eligible for the only palliative therapy. Res. J. Oncol..

[143] Farhood B., Mortezaee K., Goradel N.H., Khanlarkhani N., Salehi E., Nashtaei M.S., Najafi M., Sahebkar A. (2019). Curcumin as an anti-inflammatory agent: Implications to radiotherapy and chemotherapy. J. Cell. Physiol..

[144] Saneja A., Kumar R., Mintoo M.J., Dubey R.D., Sangwan P.L., Mondhe D.M., Panda A.K., Gupta P.N. (2019). Gemcitabine and betulinic acid co-encapsulated PLGA−PEG polymer nanoparticles for improved efficacy of cancer chemotherapy. Mater. Sci. Eng. C.

[145] Zhai J., Luwor R.B., Ahmed N., Escalona R., Tan F.H., Fong C., Ratcliffe J., Scoble J.A., Drummond C.J., Tran N. (2018). Paclitaxel-loaded self-assembled lipid nanoparticles as targeted drug delivery systems for the treatment of aggressive ovarian cancer. ACS Appl. Mater. Interfaces.

[146] Tahir N., Madni A., Balasubramanian V., Rehman M., Correia A., Kashif P.M., Mäkilä E., Salonen J., Santos H.A. (2017). Development and optimization of methotrexate-loaded lipid-polymer hybrid nanoparticles for controlled drug delivery applications. Int. J. Pharm..

[147] Xu C., Wang Y., Guo Z., Chen J., Lin L., Wu J., Tian H., Chen X. (2019). Pulmonary delivery by exploiting doxorubicin and cisplatin co-loaded nanoparticles for metastatic lung cancer therapy. J. Control. Release.

[148] Raja M.A., Arif M., Feng C., Zeenat S., Liu C.-G. (2017). Synthesis and evaluation of pH-sensitive, self-assembled chitosan-based nanoparticles as efficient doxorubicin carriers. J. Biomater. Appl..

[149] Peng X.-H., Wang Y., Huang D., Wang Y., Shin H.J., Chen Z., Spewak M.B., Mao H., Wang X., Wang Y. (2011). Targeted delivery of cisplatin to lung cancer using ScFvEGFR-heparin-cisplatin nanoparticles. ACS Nano.

[150] Zhang J., Miao L., Guo S., Zhang Y., Zhang L., Satterlee A., Kim W.Y., Huang L. (2014). Synergistic anti-tumor effects of combined gemcitabine and cisplatin nanoparticles in a stroma-rich bladder carcinoma model. J. Control. Release.

[151] Xiong X., Arvizo R.R., Saha S., Robertson D.J., McMeekin S., Bhattacharya R., Mukherjee P. (2014). Sensitization of ovarian cancer cells to cisplatin by gold nanoparticles. Oncotarget.

[152] Rauf N., Nawaz A., Ullah H., Ullah R., Nabi G., Ullah A., Wahab F., Jahan S., Fu J. (2021). Therapeutic effects of chitosan-embedded vitamin C, E nanoparticles against cisplatin-induced gametogenic and androgenic toxicity in adult male rats. Environ. Sci. Pollut. Res..

[153] Wang Y., Dou L., He H., Zhang Y., Shen Q. (2014). Multifunctional nanoparticles as nanocarrier for vincristine sulfate delivery to overcome tumor multidrug resistance. Mol. Pharm..

[154] Sun L., Chen Y., Zhou Y., Guo D., Fan Y., Guo F., Zheng Y., Chen W. (2017). Preparation of 5-fluorouracil-loaded chitosan nanoparticles and study of the sustained release in vitro and in vivo. Asian J. Pharm. Sci..

[155] Smith T., Affram K., Nottingham E.L., Han B., Amissah F., Krishnan S., Trevino J., Agyare E. (2020). Application of smart solid lipid nanoparticles to enhance the efficacy of 5-fluorouracil in the treatment of colorectal cancer. Sci. Rep..

[156] Zhong H., Mu J., Du Y., Xu Z., Xu Y., Yu N., Zhang S., Guo S. (2020). Acid-triggered release of native gemcitabine conjugated in polyketal nanoparticles for enhanced anticancer therapy. Biomacromolecules.

[157] Yalcin T.E., Ilbasmis-Tamer S., Takka S. (2020). Antitumor activity of gemcitabine hydrochloride loaded lipid polymer hybrid nanoparticles (LPHNs): In vitro and in vivo. Int. J. Pharm..

[158] Fang Y., Wang H., Dou H.J., Fan X., Fei X.C., Wang L., Cheng S., Janin A., Wang L., Zhao W.L. (2018). Doxorubicin-loaded dextran-based nano-carriers for highly efficient inhibition of lymphoma cell growth and synchronous reduction of cardiac toxicity. Int. J. Nanomed..

[159] Dai T., Zhou S., Yin C., Li S., Cao W., Liu W., Sun K., Dou H., Cao Y., Zhou G. (2014). Dextran-based fluorescent nanoprobes for sentinel lymph node mapping. Biomaterials.

[160] Cuvier C., Roblot-Treupel L., Millot J., Lizard G., Chevillard S., Manfait M., Couvreur P., Poupon M. (1992). Doxorubicin-loaded nanospheres bypass tumor cell multidrug resistance. Biochem. Pharmacol..

[161] Némati F., Dubernet C., Fessi H., de Verdiere A.C., Poupon M., Puisieux F., Couvreur P. (1996). Reversion of multidrug resistance using nanoparticles in vitro: Influence of the nature of the polymer. Int. J. Pharm..

[162] De Verdiere A.C., Dubernet C., Nemati F., Soma E., Appel M., Ferte J., Bernard S., Puisieux F., Couvreur P. (1997). Reversion of multidrug resistance with polyalkylcyanoacrylate nanoparticles: Towards a mechanism of action. Br. J. Cancer.

[163] van Vlerken L.E., Duan Z., Seiden M.V., Amiji M.M. (2007). Modulation of intracellular ceramide using polymeric nanoparticles to overcome multidrug resistance in cancer. Cancer Res..

[164] Yuan Y., Wang L., Du W., Ding Z., Zhang J., Han T., An L., Zhang H., Liang G. (2015). Intracellular self-assembly of Taxol nanoparticles for overcoming multidrug resistance. Angew. Chem. Int. Ed..

[165] Wang J., Li N., Cao L., Gao C., Zhang Y., Shuai Q., Xie J., Luo K., Yang J., Gu Z. (2020). DOX-loaded peptide dendritic copolymer nanoparticles for combating multidrug resistance by regulating the lysosomal pathway of apoptosis in breast cancer cells. J. Mater. Chem. B.

[166] Mu C.-F., Cui F., Yin Y.-M., Cho H.-J., Kim D.-D. (2020). Docetaxel-loaded chitosan-cholesterol conjugate-based self-assembled nanoparticles for overcoming multidrug resistance in cancer cells. Pharmaceutics.

[167] Jia L., Li Z., Zheng D., Li Z., Zhao Z. (2021). A targeted and redox/pH-responsive chitosan oligosaccharide derivatives based nanohybrids for overcoming multidrug resistance of breast cancer cells. Carbohydr. Polym..

[168] Ma C., Wang Z., Xu T., He Z., Wei Y. (2020). The approved gene therapy drugs worldwide: From 1998 to 2019. Biotechnol. Adv..

[169] Ahmed S., Abdelnabi A., Maguire C., Doha M., Sagers J., Lewis R., Muzikansky A., Giovannini M., Stemmer-Rachamimov A., Stankovic K. (2019). Gene therapy with apoptosis-associated speck-like protein, a newly described schwannoma tumor suppressor, inhibits schwannoma growth in vivo. Neuro-Oncol..

[170] Li H., Nakano T., Hotta A. (2014). Genetic correction using engineered nucleases for gene therapy applications. Dev. Growth Differ..

[171] Tak Y., Horng J., Perry N., Schultz H., Iyer S., Yao Q., Zou L., Aryee M., Pinello L., Joung J. (2021). Augmenting and directing long-range CRISPR-mediated activation in human cells. Nat. Methods.

[172] Guan S., Rosenecker J. (2017). Nanotechnologies in delivery of mRNA therapeutics using nonviral vector-based delivery systems. Gene Ther..

[173] Gao S., Dagnaes-Hansen F., Nielsen E.J., Wengel J., Besenbacher F., Howard K.A., Kjems J. (2009). The effect of chemical modification and nanoparticle formulation on stability and biodistribution of siRNA in mice. Mol. Ther..

[174] Liu J., Lu X., Wu T., Wu X., Han L., Ding B. (2021). Branched Antisense and siRNA Co-Assembled Nanoplatform for Combined Gene Silencing and Tumor Therapy. Angew. Chem..

[175] Yue R., Chen M., Ma N. (2020). Dual MicroRNA-triggered drug release system for combined chemotherapy and gene therapy with logic operation. ACS Appl. Mater. Interfaces.

[176] Guevara M.L., Persano F., Persano S. (2020). Advances in lipid nanoparticles for mRNA-based cancer immunotherapy. Front. Chem..

[177] Nair J., Nair A., Veerappan S., Sen D. (2020). Translatable gene therapy for lung cancer using Crispr CAS9—An exploratory review. Cancer Gene Ther..

[178] Zhou Y., Han S., Liang Z., Zhao M., Liu G., Wu J. (2020). Progress in arginine-based gene delivery systems. J. Mater. Chem. B.

[179] Ward D.M., Shodeinde A.B., Peppas N.A. (2021). Innovations in Biomaterial Design toward Successful RNA Interference Therapy for Cancer Treatment. Adv. Healthc. Mater..

[180] Karlsson J., Rui Y., Kozielski K.L., Placone A.L., Choi O., Tzeng S.Y., Kim J., Keyes J.J., Bogorad M.I., Gabrielson K. (2019). Engineered nanoparticles for systemic siRNA delivery to malignant brain tumours. Nanoscale.

[181] Li F.Q., Yu Q.L., Liu Y.H., Yu H.J., Chen Y., Liu Y. (2020). Highly efficient photocontrolled targeted delivery of siRNA by a cyclodextrin-based supramolecular nanoassembly. Chem. Commun..

[182] Kroll A.V., Fang R.H., Jiang Y., Zhou J., Wei X., Yu C.L., Gao J., Luk B.T., Dehaini D., Gao W. (2017). Nanoparticulate Delivery of Cancer Cell Membrane Elicits Multiantigenic Antitumor Immunity. Adv. Mater..

[183] Chen M., Chen M., He J. (2019). Cancer cell membrane cloaking nanoparticles for targeted co-delivery of doxorubicin and PD-L1 siRNA. Artif. Cells Nanomed. Biotechnol..

[184] Zhang C., Zhang X., Zhao W., Zeng C., Li W., Li B., Luo X., Li J., Jiang J., Deng B. (2019). Chemotherapy drugs derived nanoparticles encapsulating mRNA encoding tumor suppressor proteins to treat triple-negative breast cancer. Nano Res..

[185] Xu X., Li L., Li X., Tao D., Zhang P., Gong J. (2020). Aptamer-protamine-siRNA nanoparticles in targeted therapy of ErbB3 positive breast cancer cells. Int. J. Pharm..

[186] Izadi S., Moslehi A., Kheiry H., Kiani F.K., Ahmadi A., Masjedi A., Ghani S., Rafiee B., Karpisheh V., Hajizadeh F. (2020). Codelivery of HIF-1α siRNA and dinaciclib by carboxylated graphene oxide-trimethyl chitosan-hyaluronate nanoparticles significantly suppresses cancer cell progression. Pharm. Res..

[187] Tang Q., Liu J., Jiang Y., Zhang M., Mao L., Wang M. (2019). Cell-selective messenger RNA delivery and CRISPR/Cas9 genome editing by modulating the interface of phenylboronic acid-derived lipid nanoparticles and cellular surface sialic acid. ACS Appl. Mater. Interfaces.

[188] Xie Y., Wang Y., Li J., Hang Y., Jaramillo L., Wehrkamp C.J., Phillippi M.A., Mohr A.M., Chen Y., Talmon G.A. (2018). Cholangiocarcinoma therapy with nanoparticles that combine downregulation of MicroRNA-210 with inhibition of cancer cell invasiveness. Theranostics.

[189] Couzin-Frankel J. (2013). Cancer Immunotherapy.

[190] Dillman R.O. (2011). Cancer immunotherapy. Cancer Biother. Radiopharm..

[191] Xu X., Li T., Shen S., Wang J., Abdou P., Gu Z., Mo R. (2019). Advances in engineering cells for cancer immunotherapy. Theranostics.

[192] Byun D.J., Wolchok J.D., Rosenberg L.M., Girotra M. (2017). Cancer immunotherapy—Immune checkpoint blockade and associated endocrinopathies. Nat. Rev. Endocrinol..

[193] Fan J., Shang D., Han B., Song J., Chen H., Yang J.-M. (2018). Adoptive cell transfer: Is it a promising immunotherapy for colorectal cancer?. Theranostics.

[194] Modak S., Le Luduec J.-B., Cheung I.Y., Goldman D.A., Ostrovnaya I., Doubrovina E., Basu E., Kushner B.H., Kramer K., Roberts S.S. (2018). Adoptive immunotherapy with haploidentical natural killer cells and Anti-GD2 monoclonal antibody m3F8 for resistant neuroblastoma: Results of a phase I study. Oncoimmunology.

[195] Song W., Zhang M. (2020). Use of CAR-T cell therapy, PD-1 blockade, and their combination for the treatment of hematological malignancies. Clin. Immunol..

[196] Yang C.-Y., Fan M.H., Miao C.H., Liao Y.J., Yuan R.-H., Liu C.L. (2020). Engineering chimeric antigen receptor T cells against immune checkpoint inhibitors PD-1/PD-L1 for treating pancreatic cancer. Mol. Ther.-Oncolytics.

[197] Wang S., Sun Z., Hou Y. (2021). Engineering Nanoparticles toward the Modulation of Emerging Cancer Immunotherapy. Adv. Healthc. Mater..

[198] Abdalla A.M.E., Xiao L., Miao Y., Huang L., Fadlallah G.M., Gauthier M., Ouyang C., Yang G. (2020). Nanotechnology Promotes Genetic and Functional Modifications of Therapeutic T Cells Against Cancer. Adv. Sci. (Weinh).

[199] Shahzad K.A., Naeem M., Zhang L., Wan X., Song S., Pei W., Zhao C., Jin X., Shen C. (2020). Design and optimization of PLGA particles to deliver immunomodulatory drugs for the prevention of skin allograft rejection. Immunol. Investig..

[200] Osada A., Mangel L., Fijuth J., Żurawski B., Ursulovic T., Nikolin B., Djan I., Olson J.G. (2020). Phase IIa/IIb clinical trial of NC-6004 (Nanoparticle Cisplatin) plus Pembrolizumab in patients with head and neck cancer (HNSCC) who have failed platinum or a platinum-containing regimen. Eur. J. Cancer.

[201] Sugisaka J., Sugawara S., Toi Y., Ogasawara T., Aso M., Tsurumi K., Ono K., Shimizu H., Domeki Y., Aiba T. (2019). Pembrolizumab plus chemotherapy versus pembrolizumab monotherapy for PD-L1-positive advanced non-small cell lung cancer in the real world. Ann. Oncol..

[202] Passariello M., Camorani S., Vetrei C., Ricci S., Cerchia L., De Lorenzo C. (2020). Ipilimumab and its derived EGFR aptamer-based conjugate induce efficient NK cell activation against cancer cells. Cancers.

[203] Naves L.B., Dhand C., Venugopal J.R., Rajamani L., Ramakrishna S., Almeida L. (2017). Nanotechnology for the treatment of melanoma skin cancer. Prog. Biomater..

[204] Naito T., Shiraishi H., Fujiwara Y. (2021). Durvalumab for the treatment of PD-L1 non-small cell lung cancer. Expert Rev. Precis. Med. Drug Dev..

[205] Awasthi R., Pacaud L., Waldron E., Tam C., Jäger U., Borchmann P., Jaglowski S., Foley S., van Besien K., Wagner-Johnston N. (2020). Tisagenlecleucel cellular kinetics, dose, and immunogenicity in relation to clinical factors in relapsed/refractory DLBCL. Blood Adv..

[206] Sutherland S.I., Ju X., Horvath L., Clark G.J. (2021). Moving on From Sipuleucel-T: New Dendritic Cell Vaccine Strategies for Prostate Cancer. Front. Immunol..

[207] Habibi N., Christau S., Ochyl L.J., Fan Z., Hassani Najafabadi A., Kuehnhammer M., Zhang M., Helgeson M., Klitzing R., Moon J.J. (2020). Engineered Ovalbumin Nanoparticles for Cancer Immunotherapy. Adv. Ther..

[208] Doshi N., Swiston A.J., Gilbert J.B., Alcaraz M.L., Cohen R.E., Rubner M.F., Mitragotri S. (2011). Cell-based drug delivery devices using phagocytosis-resistant backpacks. Adv. Mater..

[209] Si J., Shao S., Shen Y., Wang K. (2016). Macrophages as Active Nanocarriers for Targeted Early and Adjuvant Cancer Chemotherapy. Small.

[210] Ochyl L.J., Bazzill J.D., Park C., Xu Y., Kuai R., Moon J.J. (2018). PEGylated tumor cell membrane vesicles as a new vaccine platform for cancer immunotherapy. Biomaterials.

[211] Wu M., Liu X., Bai H., Lai L., Chen Q., Huang G., Liu B., Tang G. (2019). Surface-Layer Protein-Enhanced Immunotherapy Based on Cell Membrane-Coated Nanoparticles for the Effective Inhibition of Tumor Growth and Metastasis. ACS Appl. Mater. Interfaces.

[212] Deng G., Sun Z., Li S., Peng X., Li W., Zhou L., Ma Y., Gong P., Cai L. (2018). Cell-Membrane Immunotherapy Based on Natural Killer Cell Membrane Coated Nanoparticles for the Effective Inhibition of Primary and Abscopal Tumor Growth. ACS Nano.

[213] Sun L., Suo C., Li S.-T., Zhang H., Gao P. (2018). Metabolic reprogramming for cancer cells and their microenvironment: Beyond the Warburg Effect. Biochim. Et Biophys. Acta-Rev. Cancer.

[214] Sen T., Tong P., Stewart C.A., Cristea S., Valliani A., Shames D.S., Redwood A.B., Fan Y.H., Li L., Glisson B.S. (2017). CHK1 inhibition in small-cell lung cancer produces single-agent activity in biomarker-defined disease subsets and combination activity with cisplatin or olaparib. Cancer Res..

[215] Yi Y., Liu Z., Fang L., Li J., Liu W., Wang F., Fu P., Xie C., Liu J., Song B. (2020). Comparison between single-agent and combination chemotherapy as second-line treatment for advanced non-small cell lung cancer: A multi-institutional retrospective analysis. Cancer Chemother. Pharmacol..

[216] Yang W., Zhou P., Liang L., Cao Y., Qiao J., Li X., Teng Z., Wang L. (2018). Nanogel-Incorporated Injectable Hydrogel for Synergistic Therapy Based on Sequential Local Delivery of Combretastatin-A4 Phosphate (CA4P) and Doxorubicin (DOX). ACS Appl. Mater. Interfaces.

[217] Zhang H., Wang G., Yang H. (2011). Drug delivery systems for differential release in combination therapy. Expert Opin. Drug Deliv..

[218] Li Y., Thambi T., Lee D.S. (2018). Co-delivery of drugs and genes using polymeric nanoparticles for synergistic cancer therapeutic effects. Adv. Healthc. Mater..

[219] Guo Z., Sui J., Ma M., Hu J., Sun Y., Yang L., Fan Y., Zhang X. (2020). pH-Responsive charge switchable PEGylated epsilon-poly-l-lysine polymeric nanoparticles-assisted combination therapy for improving breast cancer treatment. J Control. Release.

[220] Liu J., Wu M., Pan Y., Duan Y., Dong Z., Chao Y., Liu Z., Liu B. (2020). Biodegradable Nanoscale Coordination Polymers for Targeted Tumor Combination Therapy with Oxidative Stress Amplification. Adv. Funct. Mater..

[221] Wang H., Zhang C., Zhang Y., Tian R., Cheng G., Pan H., Cui M., Chang J. (2020). An efficient delivery of photosensitizers and hypoxic prodrugs for a tumor combination therapy by membrane camouflage nanoparticles. J. Mater. Chem. B.

[222] Xu C., Liu W., Hu Y., Li W., Di W. (2020). Bioinspired tumor-homing nanoplatform for co-delivery of paclitaxel and siRNA-E7 to HPV-related cervical malignancies for synergistic therapy. Theranostics.

[223] Singh S.K., Singh S., Lillard J.W., Singh R. (2017). Drug delivery approaches for breast cancer. Int. J. Nanomed..

[224] Persano F., Gigli G., Leporatti S. (2021). Lipid-polymer hybrid nanoparticles in cancer therapy: Current overview and future directions. Nano Express.

[225] Jin M., Jin G., Kang L., Chen L., Gao Z., Huang W. (2018). Smart polymeric nanoparticles with pH-responsive and PEG-detachable properties for co-delivering paclitaxel and survivin siRNA to enhance antitumor outcomes. Int. J. Nanomed..

[226] Heo M., Lim Y. (2014). Programmed nanoparticles for combined immunomodulation, antigen presentation and tracking of immunotherapeutic cells. Biomaterials.

[227] Zhang J., Tang H., Liu Z., Chen B. (2017). Effects of major parameters of nanoparticles on their physical and chemical properties and recent application of nanodrug delivery system in targeted chemotherapy. Int. J. Nanomed..

[228] Mi P., Miyata K., Kataoka K., Cabral H. (2021). Clinical translation of self-assembled cancer nanomedicines. Adv. Ther..

[229] Xing Y., Cai Y., Cheng J., Xu X. (2020). Applications of molybdenum oxide nanomaterials in the synergistic diagnosis and treatment of tumor. Appl. Nanosci..

[230] Tallury P., Malhotra A., Byrne L.M., Santra S. (2010). Nanobioimaging and sensing of infectious diseases. Adv. Drug Del. Rev..

[231] Mérian J., Gravier J., Navarro F., Texier I. (2012). Fluorescent nanoprobes dedicated to in vivo imaging: From preclinical validations to clinical translation. Molecules.

[232] Napp J., Mathejczyk J.E., Alves F. (2011). Optical imaging in vivo with a focus on paediatric disease: Technical progress, current preclinical and clinical applications and future perspectives. Pediatric Radiol..

[233] Vollrath A., Schubert S., Schubert U.S. (2013). Fluorescence imaging of cancer tissue based on metal-free polymeric nanoparticles—A review. J. Mater. Chem. B.

[234] Reisch A., Klymchenko A.S. (2016). Fluorescent polymer nanoparticles based on dyes: Seeking brighter tools for bioimaging. Small.

[235] Klymchenko A.S., Liu F., Collot M., Anton N. (2021). Dye-Loaded Nanoemulsions: Biomimetic Fluorescent Nanocarriers for Bioimaging and Nanomedicine. Adv. Healthc. Mater..

